# Association of dietary proteins with serum creatinine and estimated glomerular filtration rate in a general population sample: the CHRIS study

**DOI:** 10.1007/s40620-022-01409-7

**Published:** 2022-08-05

**Authors:** Vladimir Vukovic, Essi Hantikainen, Athina Raftopoulou, Martin Gögele, Johannes Rainer, Francisco S. Domingues, Peter P. Pramstaller, Vanessa Garcia-Larsen, Cristian Pattaro

**Affiliations:** 1 Eurac Research, Institute for Biomedicine (Affiliated to the University of Lübeck), Via Volta 21, 39100 Bolzano, Italy; 2 Department of Epidemiology, Faculty of Medicine, University of Novi Sad, Hajduk Veljkova 3, 21000 Novi Sad, Serbia; 3 Centre for Disease Control and Prevention, Institute of Public Health of Vojvodina, Novi Sad, Serbia; 4 Department of Economics, University of Patras, Patras, Greece; 5 Department of International Health, Center for Human Nutrition, Johns Hopkins Bloomberg School of Public Health, Baltimore, MD USA

**Keywords:** Kidney function, Creatinine, Glomerular filtration rate, Dietary proteins, Protein sources

## Abstract

**Background:**

Diet is known to affect kidney function. However, population-based studies provide contrasting evidence, resulting in a poor understanding of the effect of proteins from specific foods on kidney health.

**Methods:**

We analyzed the effect of total daily protein intake (TDPI) and source-specific daily protein intake (DPI) on fasting serum creatinine (SCr) and estimated glomerular filtration rate (eGFR) in the Cooperative Health Research In South Tyrol (CHRIS) cross-sectional study (*n* = 5889), using the GA^2^LEN food frequency questionnaire for TDPI and DPI estimation. We fitted multivariable adjusted mixed models of SCr and eGFR on TDPI and DPI quartiles (Q1-Q4) in the overall sample, and after removing individuals with known hypertension, diabetes or chronic kidney disease (CKD).

**Results:**

Higher TDPI as well as DPI from overall animal sources, fish, and poultry, were associated with higher SCr (trend test *p*, *p*_trend_ < 0.01), with larger effect after excluding individuals with known hypertension, diabetes or CKD. The eGFR was lower at higher TDPI (Q4 vs Q1: − 1.6 ml/min/1.73 m^2^; 95% CI − 2.5, − 0.7; *p*_trend_ = 3e−4) and DPI from fish (Q4 vs Q1: − 2.1 ml/min/1.73 m^2^; 95% CI − 2.9, − 1.20; *p*_trend_ = 4.3e−6), overall animal source (Q4 vs Q1: − 1.6 ml/min/1.73 m^2^; 95% CI −2.5, − 0.8), processed meat (Q4 vs Q1: − 1.4 ml/min/1.73 m^2^; *p*_trend_ = 0.027), red meat, offal and processed meat (Q4 vs Q1: − 1.4 ml/min/1.73 m^2^; *p*_trend_ = 0.015) and poultry (Q4 vs Q1: − 0.9 ml/min/1.73 m^2^; *p*_trend_ = 0.015).

**Conclusions:**

TDPI and DPI from specific animal sources were positively associated with SCr and negatively associated with eGFR. Lacking an alternative marker of kidney function, confounding involving muscle mass metabolism cannot be fully excluded.

**Graphical abstract:**

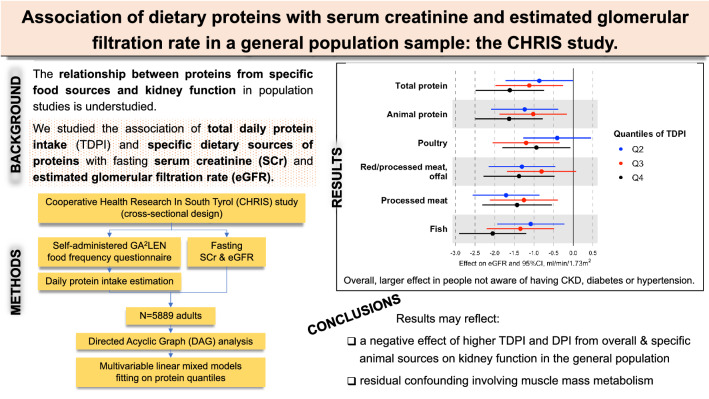

**Supplementary Information:**

The online version contains supplementary material available at 10.1007/s40620-022-01409-7.

## Introduction

Chronic kidney disease (CKD) affects > 10% of the global population [1], has a rising prevalence, limited treatment options [2] and causes substantial economic burden at the individual and social level. Western diet, characterized by high intake of animal proteins, processed foods, sugars and saturated fats [3], is a key contributor to the increased burden of non-communicable diseases, including CKD [3, 4]. The negative effect of dietary proteins on kidney health has fostered the implementation of low protein diets in the initial CKD stages [5].

Higher protein (HP) intake, implicating higher circulating amino acids, modulates the kidney hemodynamic, leading to vasodilation, which causes increased kidney blood flow and intraglomerular pressure, further leading to higher glomerular filtration rate and excretion of nitrogen end-products [6]. HP intake can also induce additional acid load to the kidneys, mostly by increasing phosphate levels and sulfate loads, it can increase ammoniagenesis and acid excretion [7]. Repeated glomerular hyperfiltration episodes resulting from frequent HP intake may contribute to structural glomerular damage in people with a reduced number of nephrons. In the long term, this can lead to GFR decline or accelerated CKD progression [6, 8], even though exceptions exist such as healthy athletes consuming high daily protein amounts without apparent kidney impairment [9].

Despite such a good rationale of structural consequences of protein-rich diets on kidney function, epidemiological studies provide contrasting evidence [10]: while some population studies in healthy adults reported associations between HP intake and estimated GFR (eGFR) decline [11], others found no evidence [12–15]. While a systematic review of clinical trials lasting > 4 weeks on 2140 CKD-free individuals observed that HP diet was associated with post intervention GFR levels [16], a more recent meta-analysis on 1358 individuals from 28 studies observed that HP diet was not associated with eGFR change over time, concluding that HP diet does not adversely affect kidney function in healthy adults [17]. Intervention studies are instrumental to test specific hypotheses, but they suffer limitations such as small sample size, short intervention periods, low compliance and uncertain generalizability of the observed effects, as the controlled setting in which they are conducted may not represent daily life complexities [18]. Observational studies can complement intervention studies by exploiting their much larger sample size to conduct hypothesis-generating analyses [18].

Given the relevance of assessing how habitual protein intake is associated with kidney function in the general population and which specific protein sources drive such associations, we investigated the effect of protein intake from different dietary sources on fasting serum creatinine (SCr) and eGFR in the Cooperative Health Research In South Tyrol (CHRIS) study [19], a cross-sectional population-based study.

## Materials and methods

### Study design

The CHRIS study [19, 20], was carried out between 2011 and 2018 in adult participants from the Vinschgau/Val Venosta community district (South Tyrol, Italy). All participants underwent blood sampling, urine collection, anthropometric analysis, and clinical examinations, in the early morning following overnight fasting. Medical history was reconstructed through interviewer- and self-administered questionnnaires.

SCr was measured using an enzymatic method (Abbott diagnostic Architect c16000 system). eGFR was estimated with the CKD-EPI formula [21] using the R package ‘nephro’ v1.2 (https://cran.r-project.org/web/packages/nephro) [22]. Given the better distribution of linear model residuals, we prioritized the analysis of SCr for model selection. Selected models were then fitted to eGFR for clinical interpretation.

Body mass index (BMI), educational level, physical activity, and smoking habits, defined in Supplementary Note 1, were considered as potential confounders. Since awareness of having hypertension (HTN), diabetes mellitus (DM), or CKD may modify dietary habits, known comorbidity was defined as at least one positive answer to the following questions: “*Has a doctor ever said that you have high blood pressure or hypertension?*”; “*Do you have diabetes mellitus?”*; and “*Has a doctor ever told you that you have a reduced kidney function or a renal failure?*”.

### Food frequency questionnaire

Dietary habits over the last 12 months were assessed using the Global Allergy and Asthma European Network (GA^2^LEN) food frequency questionnaire (FFQ) [23] allowing nutritional intake estimation. This FFQ includes 32 sections and uses standard food portion sizes following the Food Standard Agency’s Food Portion Sizes Guidelines [24]. Participants were asked to indicate how often, on average, they consumed specific quantities of the indicated items (responses ranging from rarely/never to ≥ 2 times/day). Validation of the FFQ is discussed in Supplementary Note 2. We adapted the German and Italian GA^2^LEN FFQ to include local specific foods. The FFQ was submitted to participants as described in Supplementary Note 2.

Frequency of specific food consumption was transformed into grams (g) per day for each item, including alcohol, and converted into specific nutrient estimates using the latest edition of the Composition of Foods Integrated Dataset [25]. Total energy intake (TEI, in kcal) was estimated as energy coming from proteins (4 kcal/g), fat (9 kcal/g), carbohydrates (expressed as monosaccharides; 3.75 kcal/g) and alcohol (7 kcal/g). Besides estimation of the total daily protein intake (TDPI), we attributed the specific protein sources, namely: all animal sources; all plant sources; combined sources (items containing both animal and plant proteins); legumes; grains; other plant sources (vegetable, fruit and nuts); red meat, offal, and processed meat; processed meat alone; poultry; fish; dairy; and eggs (Supplementary Table 1 contains a detailed description of all items and their corresponding protein content). TDPI and source-specific daily protein intakes (DPI) were expressed as percentage of TEI (%energy = ((protein in g × 4 kcal/g)/TEI in kcal) × 100) [26].

### Statistical analyses

We included data from all 5972 study participants to whom the FFQ was administered between May 5th, 2014 and June 16th, 2017. We excluded 77 individuals with > 20% missing FFQ items or classified in the < 0.5th or > 99.5th percentile of the TEI/basal metabolic rate ratio [27], and 6 additional participants with missing SCr values, leaving 5889 participants for analysis.

**Fig. 1:**
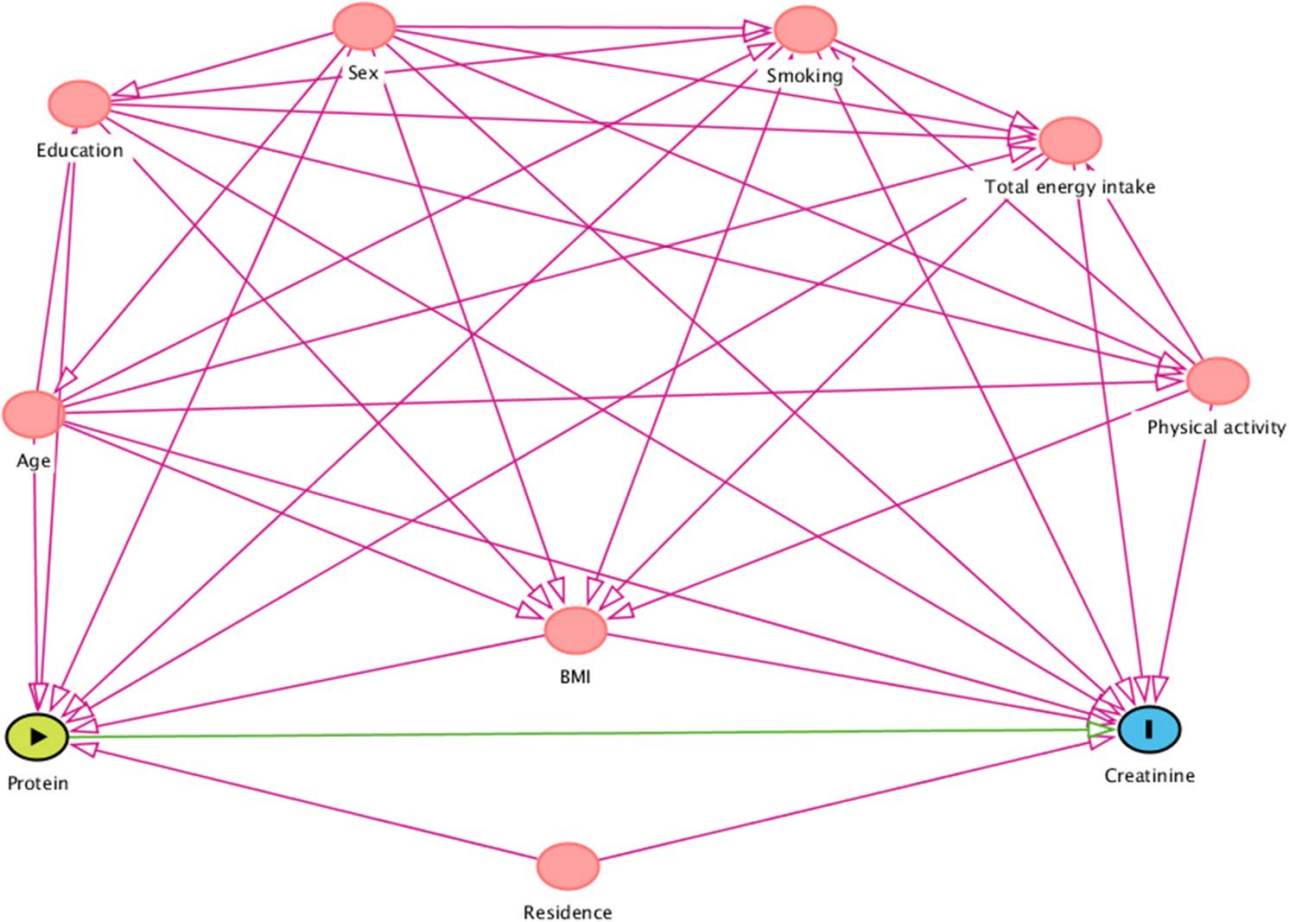
Directed acyclic graph (DAG). Arrows represent direct causal effects of one variable on another. Potential confounders are represented by red circles. Based on the assumptions from the DAG, a minimal sufficient adjustment set required for estimating the total effect of dietary protein intake on serum creatinine includes: age, sex, education, BMI, physical activity, smoking habits, total energy intake, and municipality of residence

Potential confounders were identified based on the available literature and study design features. Using directed acyclic graph (DAG) analysis (DAGitty v.3.0; http://www.dagitty.net) we assessed relationships between all involved variables to prevent over-adjustment bias [28] and to select the best variables (Fig. 1). TDPI and DPI were categorized into quartiles, setting the lowest quartile as reference category for hypothesis testing. To investigate trends, we defined for each protein source a quantitative variable with values corresponding to the within-quartile median level. To overcome skewness, continuous TDPI and DPI were ln-transformed. We fitted four linear mixed regression models (LMMs) to assess the presence of a linear association between SCr and TDPI and source-specific DPI:

- Model 1: adjusted for age, sex, TEI and municipality of residence;
- Model 2: further adjusting for BMI, physical activity, education, and smoking status;
- Model 3: same as Model 2 but excluding 1412 (24.2%) individuals with known HTN, DM or CKD.

Model 3 was fitted also to eGFR and submitted to five sensitivity analyses: (i) excluding individuals on a special diet (for weight loss or disease management) at the time of participation or not answering such question (5%); (ii) excluding individuals (8%) with missing physical activity data; (iii) adjusting for dietary sodium intake (g/day, estimated from the FFQ and adjusted for TEI using the nutrient residual method [26]) given its influence on kidney function and oxygenation [29]; (iv) adjusting for fasting status; (v) adjusting for each of the other sources of protein. Significance level was set at 0.05. Supplementary Note 3 includes additional details on statistical models and software used.

## Results

Participants’ mean age was 45.9 years (standard deviation, SD = 16.1), with 54.9% females (Table 1). Mean TDPI was 83.9 g/day (SD = 28.7), increasing from 70 g/day in Q1 to 97.4 g/day in Q4. Mean TDPI expressed per kg body weight (g/kg/day) was 1.2 g/kg (SD = 0.4) and increased from 1.0 g/kg (SD = 0.4) in Q1 to 1.3 g/kg (SD = 0.5) in Q4. Mean TEI was 1950 kcal/day (SD = 607), decreasing along with increasing TDPI quartiles. Mean TDPI contribution to TEI was 17.2% (SD = 2.5%). Overall animal sources contributed more to TDPI (10.8%, SD = 2.9%) than overall plant sources (overall 5.2%, SD = 1.3%), and increased from 7.6% to 14.2% through TDPI quartiles, whereas DPI from plant sources was stable across TDPI quartiles. The specific protein sources mostly contributing to TEI were: red meat, offal and processed meat; dairy; grains; fish; poultry. Contribution of other protein sources to TEI was < 1%. SCr (mean = 0.86 mg/dL, SD = 0.14) increased at increasing TDPI quartiles. Correspondingly, eGFR (mean = 93.3 ml/min/1.73 m^2^, SD = 15.6) decreased, as expected. Thirty-one (0.5%), 149 (2.5%) and 1348 (23%) individuals reported CKD, DM, and HTN, respectively. Age and percent of females and hypertensive individuals decreased with increasing TDPI quartiles, while BMI increased.

**Table 1:** Characteristics of the 5889 study participants by quartiles of the total daily protein intake (TDPI) expressed in % energy

Characteristics	TDPI quartiles				Overall
	Q1	Q2	Q3	Q4	
Sample size	(*N* = 1473)	(*N* = 1472)	(*N* = 1472)	(*N* = 1472)	(*N* = 5889)
*TDPI in %energy*					
Mean (SD)	14.1 (1.27)	16.4 (0.4)	17.9 (0.5)	20.5 (1.6)	17.2 (2.5)
Range (min, max)	7.6, 15.6	15.7, 17.1	17.2, 18.8	18.9, 34.4	7.6, 34.4
Age (years), mean (SD)	49.4 (16.4)	46.4 (15.9)	45.8 (16.0)	42.1 (15.4)	45.9 (16.1)
BMI (kg/m^2^), mean (SD)	25.4 (4.3)	25.9 (4.5)	26.3 (4.7)	26.5 (4.9)	26.0 (4.6)
Serum creatinine (mg/dL), mean (SD)	0.86 (0.14)	0.86 (0.14)	0.87 (0.14)	0.87 (0.14)	0.86 (0.14)
eGFR (ml/min/1.73 m^2^), mean (SD)	91.4 (16.0)	92.9 (15.8)	93.1 (15.7)	95.7 (15.7)	93.3 (15.9)
TDPI (g/day), mean (SD)	70.0 (23.7)	81.2 (23.9)	87.0 (26.9)	97.4 (32.3)	83.9 (28.7)
TDPI (g/kg/day), mean (SD)	1.0 (0.4)	1.1 (0.4)	1.2 (0.4)	1.3 (0.5)	1.2 (0.4)
*DPI (%energy/day), mean (SD)*					
From animal source	7.6 (1.8)	9.9 (1.2)	11.4 (1.3)	14.2 (2.2)	10.8 (2.9)
From plant source	5.2 (1.3)	5.1 (1.1)	5.2 (1.2)	5.2 (1.3)	5.2 (1.3)
From combined sources	1.2 (0.6)	1.2 (0.5)	1.1 (0.5)	1.0 (0.5)	1.1 (0.5)
From poultry	0.6 (0.6)	1.0 (0.7)	1.1 (0.7)	1.6 (1.3)	1.1 (0.9)
From red, processed meat, offal	2.7 (1.4)	3.7 (1.4)	4.3 (1.7)	5.8 (2.6)	4.1 (2.1)
From processed meat	0.8 (0.7)	1.1 (0.8)	1.3 (0.9)	1.5 (1.1)	1.2 (0.9)
From fish	0.9 (0.7)	1.3 (0.7)	1.5 (0.8)	1.9 (1.4)	1.4 (1.0)
From eggs	0.2 (0.2)	0.2 (0.3)	0.3 (0.3)	0.3 (0.4)	0.3 (0.3)
From dairy	3.2 (1.5)	3.8 (1.5)	4.3 (1.7)	4.6 (2.0)	4.0 (1.8)
From grains	3.0 (1.1)	3.0 (1.0)	3.1 (1.1)	3.1 (1.2)	3.1 (1.1)
From legumes	0.3 (0.4)	0.3 (0.3)	0.3 (0.3)	0.3 (0.5)	0.3 (0.4)
From other plants	1.9 (0.8)	1.8 (0.8)	1.8 (0.7)	1.8 (0.8)	1.8 (0.8)
Total daily carbohydrate intake (%energy/day), mean (SD)	43.3 (6.1)	41.8 (5.0)	40.2 (5.0)	37.8 (5.2)	40.8 (5.7)
Total daily fat intake (%energy/day), mean (SD)	39.8 (5.7)	39.6 (4.8)	39.9 (4.7)	39.7 (4.7)	39.7 (5.0)
Total daily energy intake (TDEI) (kcal/day), mean (SD)	1980 (639)	1980 (581)	1940 (599)	1900 (604)	1950 (607)
*Sex, n (%)*					
Male	860 (58.4)	827 (56.2)	798 (54.2)	746 (50.7)	3231 (54.9)
Female	613 (41.6)	645 (43.8)	674 (45.8)	726 (49.3)	2658 (45.1)
*Educational level, n (%)*					
Up to lower secondary education	428 (29.0)	398 (27.0)	402 (27.3)	341 (23.2)	1569 (26.6)
Professional qualification	621 (42.2)	587 (39.9)	588 (40.0)	633 (43.0)	2429 (41.3)
Upper secondary education	297 (20.2)	341 (23.2)	331 (22.5)	351 (23.8)	1320 (22.4)
University degree or higher	127 (8.6)	144 (9.8)	149 (10.1)	143 (9.7)	563 (9.6)
Missing	0 (0)	2 (0.1)	2 (0.1)	4 (0.3)	8 (0.1)
*Physical activity (IPAQ score), n (%)*					
Low	238 (16.2)	227 (15.4)	229 (15.6)	226 (15.4)	920 (15.6)
Moderate	416 (28.2)	397 (27.0)	385 (26.1)	352 (23.9)	1550 (26.3)
High	720 (48.9)	738 (50.1)	735 (49.9)	779 (52.9)	2972 (50.5)
Missing	99 (6.7)	110 (7.5)	123 (8.4)	115 (7.8)	447 (7.6)
*Smoking habit, n (%)*					
Never smoker	840 (57.0)	874 (59.4)	770 (52.3)	765 (52.0)	3249 (55.2)
Past smoker	425 (28.9)	389 (26.4)	422 (28.7)	396 (26.9)	1632 (27.7)
Current smoker	202 (13.7)	205 (13.9)	270 (18.3)	301 (20.4)	978 (16.6)
Missing	6 (0.4)	4 (0.3)	10 (0.7)	10 (0.7)	30 (0.5)
*Special diet, n (%)*					
Yes	48 (3.3)	45 (3.1)	66 (4.5)	97 (6.6)	256 (4.4)
No	1414 (96.0)	1409 (95.7)	1391 (94.5)	1359 (92.3)	5573 (94.6)
Missing	11 (0.7)	18 (1.2)	15 (1.0)	16 (1.1)	60 (1.0)
*HTN awareness, n (%)*					
Yes	361 (24.5)	358 (24.3)	330 (22.4)	299 (20.3)	1348 (22.9)
No	1106 (75.1)	1108 (75.3)	1128 (76.6)	1165 (79.2)	4507 (76.5)
Missing	6 (0.4)	6 (0.4)	14 (1.0)	8 (0.5)	34 (0.6)
*DM awareness, n (%)*					
Yes	40 (2.7)	24 (1.7)	41 (2.8)	44 (3.0)	149 (2.5)
No	1430 (97.1)	1446 (98.2)	1430 (97.1)	1424 (96.7)	5730 (97.3)
Missing	3 (0.2)	2 (0.1)	1 (0.1)	4 (0.3)	10 (0.2)
*CKD awareness, n (%)*					
Yes	10 (0.7)	5 (0.3)	9 (0.6)	7 (0.5)	31 (0.5)
No	1457 (98.9)	1461 (99.3)	1458 (99.1)	1463 (99.4)	5839 (99.2)
Missing	6 (0.4)	6 (0.4)	5 (0.3)	2 (0.1)	19 (0.3)
*Comorbidity awareness*^*a*^*, n (%)*					
Yes	379 (25.7)	369 (25.1)	350 (23.8)	324 (22.0)	1422 (24.1)
No	1094 (74.3)	1103 (74.9)	1122 (76.2)	1148 (78.0)	4467 (75.9)

*DPI* daily protein intake, *TDPI* total DPI, *SD* standard deviation, *BMI* body mass index, *IPAQ *International Physical Activity Questionnaire, *HTN* hypertension, *DM* diabetes mellitus, *CKD* chronic kidney disease

^a^Comorbidity awareness was defined as self-reported presence of diagnosed HTN, DM or CKD

Age-, sex- and TEI-adjusted LMMs (Model 1) showed a positive relationship between TDPI quartiles and SCr (*p*_trend_ = 0.003; Table 2). Fitting a second model that accounted for lifestyle factors (Model 2) confirmed previous results (Table 2). Additional exclusion of participants with known HTN, DM or CKD (Model 3) yielded a slightly stronger association with TDPI: with increasing effect for individuals in Q4 (coefficient of association, *beta* (*b*) from 0.012 (Model 1) to 0.015 (Model 3; Table 2).

**Table 2:** Association between TDPI (expressed in %energy) and serum creatinine

	Model 1 (*n* = 5889)	Model 2 (*n* = 5841)	Model 3 (*n* = 4429)
	*b* (95% CI)	*b* (95% CI)	*b* (95% CI)
*Total daily protein intake (%energy)* *Quantiles and within-quantile ranges*			
Q1 (7.6, 15.6)	Ref.	Ref.	Ref.
Q2 (15.7, 17.1)	0.007 (− 0.001, 0.015)	0.007 (− 0.001, 0.015)	0.008 (0.0000002, 0.016)
Q3 (17.2, 18.8)	0.010 (0.002, 0.018)	0.010 (0.002, 0.018)	0.010 (0.002, 0.018)
Q4 (18.9, 34.4)	0.012 (0.004, 0.020)	0.013 (0.005, 0.021)	0.015 (0.007, 0.023)
*p for trend*^a^	0.003	0.002	5e−04
Age (years)	0.001 (0.001, 0.001)	0.001 (0.001, 0.001)	0.001 (0.0002, 0.0007)
Sex (female vs male)	− 0.164 (− 0.170, 0.158)	− 0.166 (− 0.172, − 0.160)	− 0.162 (− 0.168, − 0.156)
Total daily energy (kcal) × 1000	− 0.002 (− 0.007, 0.002)	− 0.002 (− 0.007, 0.002)	− 0.002 (− 0.007, 0.003)
BMI (kg/m^2^)		0.0003 (− 0.0003, 0.0010)	0.0004 (− 0.0003, 0.0012)
*Physical activity (IPAQ score)*			
Low		Ref.	Ref.
Moderate		− 0.009 (− 0.018, 0.000)	− 0.002 (− 0.012, 0.007)
High		− 0.010 (− 0.018, − 0.002)	0.002 (− 0.007, 0.010)
Missing		− 0.010 (− 0.022, 0.003)	− 0.004 (− 0.016, 0.009)
*Education*			
Up to lower secondary education		Ref.	Ref.
Professional qualification		− 0.002 (− 0.009, 0.005)	− 0.002 (− 0.010, 0.006)
Upper secondary education		0.016 (0.007, 0.025)	0.012 (0.003, 0.021)
University degree or higher		0.008 (− 0.004, 0.019)	0.009 (− 0.002, 0.020)
*Smoking habit*			
Never smoker		Ref.	Ref.
Past smoker		− 0.006 (− 0.012, 0.001)	− 0.006 (− 0.013, 0.001)
Current smoker		− 0.017 (− 0.026, − 0.009)	− 0.013 (− 0.021, − 0.005)

Results of the linear mixed model fitting of serum creatinine on the quartiles of TDPI in %energy

Model 1 was adjusted for: age, sex, TEI and municipality of residence; Model 2 was adjusted for: variables of Model 1 + BMI, physical activity level, educational level, and smoking habit; Model 3 corresponds to Model 2 with exclusion of individuals with comorbidity awareness

^a^The trend was tested by fitting the models with implementing a quantitative variable based on the median values of the distribution of each protein source quartile

*TDPI* total daily protein intake, *IPAQ* International Physical Activity Questionnaire, *b* coefficient of association, *CI* confidence interval, *Ref.* reference category

**Fig. 2:**
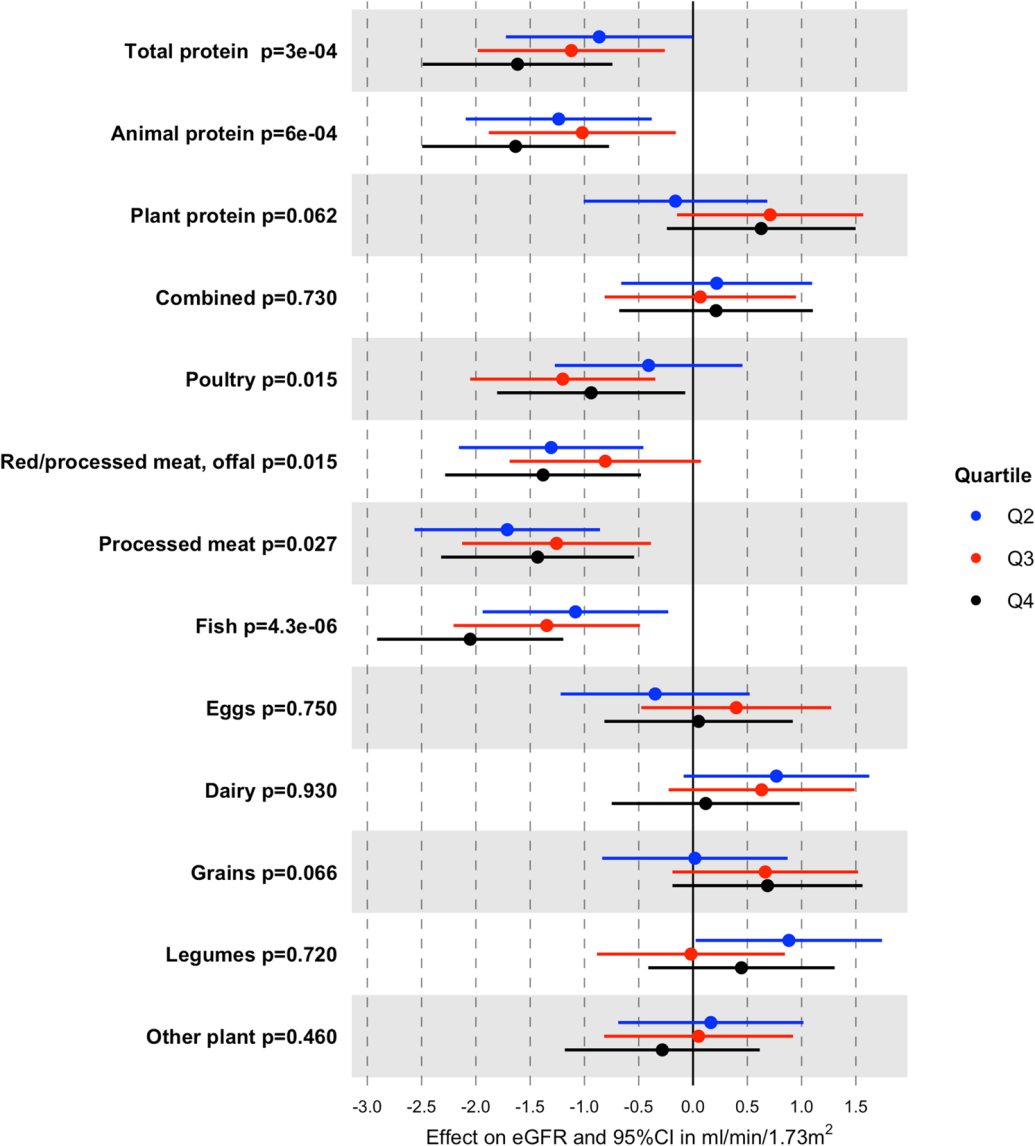
Association between quartiles of total and specific sources of protein intake and eGFR in individuals without known comorbidities. Models were adjusted for age, sex, TEI and municipality of residence, BMI, physical activity level, educational level and smoking status

Gradual exploration from Model 1 to Model 3 was conducted also for the specific protein sources (Models 1, 2 and 3 are presented in Table 3). When fitting Model 1, the most commonly associated protein sources were overall animal sources (*p*_trend_ = 0.013), poultry (*p*_trend_ = 5e−04), and fish (*p*_trend_ = 1e−05), showing that greater consumption of protein from these sources was related to increased levels of SCr. Additionally, SCr was associated with DPI from processed meat, even if no significant trend was observed (*p*_trend_ = 0.110). When comparing Models 1, 2, and 3, we observed that the association between protein from overall animal sources and SCr became even stronger in Model 3 (Model 1: Q4 *b* = 0.010, Table 3; Model 3: Q4 *b* = 0.014, Fig. 2). A similar stronger association was found with processed meat (Model 1: Q4 *b* = 0.010, Table 3; Model 3: Q4 *b* = 0.014, Fig. 2). While remaining significant, the effect of proteins from poultry on SCr was smaller as compared to Models 1 and 2. The association with protein from fish remained stable across models, except for a clearer pattern with increasing SCr levels at increasing quartiles in Model 3: *b* = 0.009, 0.012, and 0.019 for quartiles 2, 3, and 4 versus 1, respectively (Fig. 2).

**Table 3:** Association between serum creatinine and different sources of DPI (expressed in %energy): results from the three linear mixed models of serum creatinine on DPI quartiles

Protein source	Model^b^	Source-specific DPI quartiles			*p* for trend^a^	
		Q2 vs Q1	Q3 vs Q1	Q4 vs Q1		
		*b* (95% CI)	*b* (95% CI)	*b* (95% CI)		
Animal protein	1	**0.009 (0.002, 0.018)**	**0.012 (0.004, 0.021)**	**0.010 (0.002, 0.018)**	**0.013**	
	2	**0.009 (0.001, 0.017)**	**0.013 (0.005, 0.021)**	**0.011 (0.003, 0.02)**	**0.005**	
	3	**0.011 (0.002, 0.019)**	0.008 (− 0.0004**, **0.016)	**0.014 (0.006, 0.022)**	**0.002**	
Plant protein	1	0.002 (− 0.006**, **0.01)	− 0.006 (− 0.014**, **0.002)	− 0.003 (− 0.012**, **0.005)	0.210	
	2	0.001 (− 0.007**, **0.009)	− 0.007 (− 0.015**, **0.001)	− 0.005 (− 0.013**, **0.003)	0.110	
	3	0.001 (− 0.007**, **0.009)	− 0.008 (− 0.016**, **0.001)	− 0.005 (− 0.013**, **0.003)	0.110	
Combined sources	1	− 0.002 (− 0.01**, **0.006)	− 0.006 (− 0.014**, **0.002)	− 0.001 (− 0.009**, **0.008)	0.800	
	2	− 0.003 (− 0.011**, **0.005)	− 0.006 (− 0.014**, **0.003)	− 0.001 (− 0.009**, **0.007)	0.800	
	3	− 0.003 (− 0.011**, ** 0.005)	− 0.003 (− 0.011**, ** 0.005)	− 0.002 (− 0.010**, ** 0.006)	0.670	
Poultry	1	**0.009 (0.001, 0.017)**	**0.011 (0.003, 0.019)**	**0.014 (0.006, 0.022)**	**5e−04**	
	2	**0.009 (0.001, 0.017)**	**0.012 (0.004, 0.02)**	**0.015 (0.006, 0.023)**	**4e−04**	
	3	0.005 (− 0.003, 0.013)	**0.012 (0.003, 0.02)**	**0.01 (0.001, 0.018)**	**0.010**	
Red, processed meat and offal	1	0.011 (0.003, 0.019)	0.01 (0.002, 0.018)	0.006 (− 0.002, 0.015)	0.250	
	2	0.01 (0.002, 0.018)	0.01 (0.002, 0.019)	0.007 (− 0.002, 0.015)	0.210	
	3	**0.011 (0.003, 0.019)**	0.007 (− 0.001, 0.016)	**0.012 (0.003, 0.02)**	**0.028**	
Processed meat	1	0.015 (0.007, 0.023)	0.012 (0.004, 0.021)	0.01 (0.002, 0.019)	0.110	
	2	0.015 (0.007, 0.023)	0.013 (0.005, 0.021)	0.011 (0.003, 0.02)	0.076	
	3	**0.015 (0.007, 0.024)**	**0.01 (0.002, 0.019)**	**0.014 (0.005, 0.022)**	**0.023**	
Fish	1	**0.009 (0.001, 0.017)**	**0.009 (0.001, 0.017)**	**0.019 (0.010, 0.027)**	**1e−05**	
	2	**0.010 (0.002, 0.018)**	**0.009 (0.001, 0.018)**	**0.019 (0.011, 0.027)**	**1e−05**	
	3	**0.009 (0.001, 0.017)**	**0.012 (0.004, 0.02)**	**0.019 (0.010, 0.027)**	**1e−05**	
Eggs	1	− 0.003 (− 0.011**, **0.005)	− 0.006 (− 0.014**, **0.002)	− 0.001 (− 0.009**, **0.007)	0.970	
	2	− 0.003 (− 0.011**, **0.005)	− 0.006 (− 0.014**, **0.002)	− 0.002 (− 0.01**, **0.006)	0.840	
	3	0.002 (− 0.006**, **0.011)	− 0.006 (− 0.014**, **0.003)	− 0.001 (− 0.01**, **0.007)	0.630	
Dairy	1	− 0.004 (− 0.012**, **0.004)	− 0.004 (− 0.012**, **0.004)	− 0.002 (− 0.01**, **0.006)	0.700	
	2	− 0.005 (− 0.013**, **0.003)	− 0.004 (− 0.012**, **0.004)	− 0.001 (− 0.009**, **0.007)	0.900	
	3	− 0.008 (− 0.016**, **0.001)	− 0.007 (− 0.015**, **0.001)	− 0.003 (− 0.011**, **0.005)	0.560	
Grains	1	0.005 (− 0.003**, **0.013)	− 0.002 (− 0.01**, **0.006)	− 0.002 (− 0.01**, **0.006)	0.310	
	2	0.004 (− 0.004**, **0.012)	− 0.003 (− 0.011**, **0.005)	− 0.004 (− 0.012**, **0.004)	0.160	
	3	0.0003 (− 0.008**, **0.008)	− 0.006 (− 0.014**, **0.003)	− 0.005 (− 0.013**, **0.003)	0.130	
Legumes	1	− 0.006 (− 0.014**, **0.002)	0.002 (− 0.006**, **0.01)	− 0.002 (− 0.01**, **0.006)	0.950	
	2	− 0.007 (− 0.015**, **0.001)	0.002 (− 0.006**, **0.01)	− 0.002 (− 0.01**, **0.006)	0.970	
	3	− 0.009 (− 0.017**, **− 0.001)	0 (− 0.009**, **0.008)	− 0.004 (− 0.012**, **0.004)	0.740	
Other plants	1	0.003 (− 0.005**, **0.011)	− 0.001 (− 0.009**, **0.007)	0.003 (− 0.005**, **0.012)	0.580	
	2	0.002 (− 0.006**, **0.011)	− 0.001 (− 0.009**, **0.007)	0.003 (− 0.006**, **0.011)	0.680	
	3	− 0.002 (− 0.010**, ** 0.006)	− 0.002 (− 0.010**, ** 0.006)	0.003 (− 0.006**, ** 0.011)	0.440	

Models with a statistically significant *p* for trend, *p* <0.05 (in bold)

*DPI* daily protein intake, *IPAQ* International Physical Activity Questionnaire, *b* coefficient of association, *CI* confidence interval

^a^The trend was tested by fitting the models with implementing a continuous variable based on the median values of the distribution of each protein source quartile

^b^Model 1 was adjusted for: age, sex, TEI and municipality of residence; Model 2 was adjusted for: variables of Model 1 + BMI, physical activity level, educational level, and smoking habit; Model 3 corresponds to Model 2 with exclusion of individuals with comorbidity awareness

Based on these results, we used Model 3 to model the association between TDPI, DPI and eGFR (Fig. 2). Compared to individuals in Q1 of TDPI, those in higher quartiles showed lower eGFR levels (*p*_trend_ = 3e-04), with the largest effect observed for individuals in Q4 vs Q1 (*b *= − 1.6 ml/min/1.73 m^2^; 95% CI − 2.5, − 0.7). When looking at the specific protein sources, we observed the strongest association with proteins from fish, again with a clear dosage effect, with lower eGFR levels at increasing quartiles (*p*_trend_ = 4.3e−06) and the strongest association was observed for individuals in Q4 vs Q1 (*b* = − 2.1 ml/min/1.73 m^2^; 95% CI − 2.9, − 1.2). For proteins of overall animal sources, we also observed the largest effect for individuals in Q4 vs Q1 (*b* = − 1.6 ml/min/1.73 m^2^; 95% CI − 2.5, − 0.8, *p*_trend_ = 6e− 04), however with a less pronounced pattern (similar effects for Q2 and Q3). For proteins from processed meat alone, we observed significant but very similar effects for individuals across all quartiles compared to Q1 (*b* = − 1.7, − 1.3, and − 1.4 ml/min/1.73 m^2^ for Q2, Q3, and Q4, respectively; *p*_trend_ = 0.027). A similar pattern across quartiles was also observed for proteins originating from overall red meat, offal and processed meat (*b* = − 1.3, − 0.8, and − 1.4 ml/min/1.73 m^2^ for Q2, Q3, and Q4, respectively, *p*_trend_ = 0.015) and poultry (*b* = − 0.4, − 1.2, and − 0.9 ml/min/1.73 m^2^ for Q2, Q3, and Q4, respectively, *p*_trend_ = 0.015).

Results were confirmed when fitting Model 3 with continuous variables ln(TDPI %energy) and ln(DPI %energy) (Supplementary Table 2), further highlighting the negative association between TDPI as well as proteins from fish, poultry, red processed meat and offal, and processed meat alone and the eGFR (Supplementary Fig. 1). Results did not change when we excluded individuals on a special diet or with missing physical activity information or when additionally adjusting for dietary sodium intake or fasting status (Supplementary Figs. 2 to 5). Sensitivity analyses using ln(TDPI %energy) and ln(DPI %energy) as continuous variables (Supplementary Table 3) showed that adjusting for sodium intake enhanced the effect of processed meat on eGFR from − 1.4 to − 2.0 ml/min/1.73 m^2^, probably because higher consumption of processed meat is accompanied by a higher sodium intake. Modeling all protein sources together (Supplementary Table 3; Supplementary Fig. 6) yielded similar results for overall animal protein and proteins from fish, and attenuated effects for protein from poultry, red meat, offal and processed meat, and processed meat alone.

## Discussion

In a large population sample, we observed that higher total protein intake assessed through a food frequency questionnaire was associated with higher SCr levels and consequently lower eGFR. Specifically, higher intake of total animal proteins and proteins from fish, poultry, red meat, offal and processed meat were associated with lower eGFR. These associations were particularly pronounced in individuals who reported never having had a diagnosis of hypertension, diabetes or CKD.

Epidemiological evidence concerning patients with moderate to severe kidney function impairment [30, 31] or patients at risk of developing CKD [32], suggests that reducing total protein intake might preserve kidney function, with the source of protein possibly playing a role. Reducing non-dairy animal protein or consuming proteins from dairy or vegetables has been associated with less kidney function impairment [12, 33]. The Modification of Diet in Renal Disease study found that reducing dietary proteins exerted a small benefit on renal disease only for patients with moderate renal insufficiency [34]. Our focus was on individuals with normal kidney function where evidence from epidemiological studies is modest. In line with our findings, a Korean general-population study reported an association between higher total protein intake and faster eGFR decline [11]. The Atherosclerosis Risk In Communities study did not find evidence of an association between total or animal protein intake and CKD risk, but did find a negative association with a higher intake of vegetable proteins [13]. No association between total protein intake and kidney function was found in the Singapore Chinese Health study [14], Cardiovascular Health Study [15] and Nurses’ Health Study [12] among healthy individuals.

To the best of our knowledge, this is the first study investigating the relationship between habitual protein intake from a large variety of specific dietary sources, fasting SCr and eGFR in the general population. Our findings suggest that not only a higher habitual intake of total and animal protein is associated with lower kidney function, but specifically a higher intake of proteins from fish, poultry, red meat and processed meat. Results further suggest that individuals may alter their food habits and especially red meat consumption when aware of having a disease such as CKD, diabetes or hypertension.

The mechanisms underlying a potentially harmful effect of consumption of animal protein sources on kidney function are not fully understood. Animal sourced proteins might cause high acidic conditions when metabolized, whereas vegetable sourced proteins yield lower acidic load [35]. High dietary acidic load may invoke kidney adaptive mechanisms, such as increased acid excretion, thereby promoting renal injury [36]. Red meat and non-red meat proteins yield comparable acid production [36], which could explain our similar findings for red meat and poultry. The amino acid profile of different protein sources may also play a role, as previously demonstrated for hypertension [37]. Although the effect of plant and animal proteins on the background amino acid metabolism remains largely unexplored, this might explain the impact of dietary protein sources on numerous functions and health outcomes [38]. Nevertheless, dietary protein sources vary in their non-protein components, which might partially explain differential health effects found between studies investigating protein and protein-rich food sources [13]. For instance, protein-rich meals increase glucagon secretion which, in the short term, stimulates urea excretion through hyperfiltration causing GFR increase and urea reabsorption reduction and, in the long term, might become dysfunctional for the kidney [39].

An alternative explanation for the observed positive association between protein intake and SCr may reflect a better nutritional status and higher muscle mass percent in individuals with higher dietary protein intake. In contrast, elderly individuals with lower muscle mass might appear to have higher eGFR levels because of lower creatine and, consequently, creatinine production [40]. Also, specifically animal protein sources may contribute to a short-term increase in exogenous creatinine, resulting in higher total SCr and lower eGFR. Consumption of a cooked meat meal can increase SCr, even leading to misclassification of CKD patients [41]. Since the effect of cooked meat on SCr was seen to disappear after 12 hours, using fasting SCr to assess kidney function if no other markers are available has been suggested [41]. Given that blood sampling in the CHRIS study was performed in the early morning after overnight fasting, an acute effect of meat consumption on SCr levels might be negligible.

After excluding participants with known diabetes, hypertension or CKD as they might have intentionally changed their diet as part of the treatment, the associations between higher protein intake from the previously described sources and lower eGFR levels were confirmed and often became stronger. Such a stronger effect might be a consequence of removing cases of low protein consumption because of an underlying disease. The exclusion of diabetic individuals may have implicated the removal of individuals with glomerular hyperfiltration, which is often observed in the presence of diabetes. The exclusion of hypertensive participants could have led to removing cases of hemodynamic changes due to antihypertensive drugs that may have influenced the biochemical measurements.

The main strength of our study was the large population-based sample with a high percentage of complete responses to the GA^2^LEN FFQ, which was validated and considered to be appropriate to estimate average dietary intake [23]. Importantly, results were robust to several sensitivity analyses.

Several limitations should be highlighted. The cross-sectional nature of the study prevented us from sorting exposure (diet) and outcome (kidney function) chronologically, and assessing dietary effects on eGFR decline. A key limitation of the study is the lack of measured GFR. Measuring GFR is burdensome and unfeasible in large population-based studies. For this reason, we estimated GFR based on SCr. Although this is common practice in clinical and observational studies, creatinine-based eGFR may overestimate GFR in elderly people with low muscle mass. In the absence of biomarkers such as cystatin C that are independent of muscle mass metabolism, we cannot exclude that the observed associations may partially reflect the muscle mass metabolism rather than being exclusive to renal function. Other biomarkers that might have been helpful to identify specific pathways, such as intrarenal nitrogen conserving mechanisms implicated by the causal effect of dietary protein intake on renal urea excretion [42], which would require 24-h urinary urea excretion analysis, were not available. Even though we carefully controlled for confounding through an accurate DAG analysis and several sensitivity analyses, the nature of our study prevents us from making causal statements. A potential source of bias could arise from estimating protein intake through a FFQ, which is unavoidably prone to recall bias, as individuals are asked to report their average dietary intake retrospectively. Furthermore, personal characteristics, such as obesity and disease status, may affect food reporting [43]. By assessing diet and health status at the same time, differential misclassification of the exposure with an unpredicted direction of the bias might have occurred [44]. Moreover, although we measured several lifestyle factors in a detailed way, we cannot rule out unobserved residual confounding. Finally, regarding the generalizability of the results, we must point out that dietary habits in rural Alpine areas might differ from large urban centers. Our analysis involved 5889 individuals out of ~ 32,000 resident adults. Data corresponded to an intermediate release of the CHRIS study dataset for which precise participation rate figures are not available. For the CHRIS study globally, we estimated ~ 40% participation rate, well-matching the age and sex distribution of the reference population until the age of 65 (Laura Barin, Eurac Research, personal communication).

In conclusion, higher intake of proteins from animal sources such as fish, poultry, red meat, and processed meat was associated with higher SCr and lower eGFR levels. This might reflect a negative subclinical effect of those protein sources on kidney function as well as an effect on serum creatinine levels through muscle mass metabolism. Confirmation of these findings by longitudinal and causal inference studies would support the opportunity to raise awareness towards the potential negative subclinical effect of animal sourced proteins on kidney function in the general population.

## Supplementary Information

Below is the link to the electronic supplementary material.

Supplementary file1 (DOCX 2426 KB)

Supplementary file2 (XLSX 23 KB)

## Data Availability

The datasets generated and/or analyzed during the current study are available from the corresponding author on reasonable request.
