# Supplementary material for: Association of dietary proteins with serum creatinine and estimated glomerular filtration rate in a general population sample: the CHRIS study
Source: J Nephrol. 2022 Aug 5;36(1):103–14. doi: 10.1007/s40620-022-01409-7 (PMC9894942; doi:10.1007/s40620-022-01409-7)

**Supplementary Material**

Contents

[**Supplementary Note 1**. Definition of additional exposure variables 2](#_Toc100334203)

[**Supplementary Note 2**. The GA2LEN food frequency questionnaire (FFQ) 2](#_Toc100334204)

[**Supplementary Note 3**. Additional details on statistical models and software 3](#_Toc100334205)

[**Supplementary Table 1**. Food items and the amounts of containing proteins. 4](#_Toc100334206)

[**Supplementary Table 2.** Results of the linear mixed model fitting to assess the association between eGFR and ln-TDPI% and source-specific ln-DPI% (Model 3, n=4429). 4](#_Toc100334207)

[***Supplementary* Table 3.** Sensitivity analyses and corresponding results of the linear mixed model fitting to assess the association between eGFR and ln-TDPI% and source-specific ln-DPI%. 5](#_Toc100334208)

[**Supplementary Figure 1.** Association between daily total protein intake and protein from different sources and age-and sex-adjusted eGFR levels 6](#_Toc100334209)

[**Supplementary Figure 2**. Sensitivity analysis 1: Model 3 (adjusted for age, sex, TEI, municipality of residence, BMI, physical activity level, educational level, smoking habit, and excluding individuals with comorbidity awareness), further excluding individuals on a special diet* (n=4238). 7](#_Toc100334210)

[**Supplementary Figure 3.** Sensitivity analysis 2: Model was adjusted for age, sex, TEI, municipality of residence, BMI, physical activity level, educational level, smoking habit; excluding individuals with comorbidity awareness and those with missing physical activity information (n=4080). 8](#_Toc100334211)

[**Supplementary Figure 4.** Sensitivity analysis 3: Model was adjusted for age, sex, TEI, municipality of residence, BMI, physical activity level, educational level, smoking habit; excluding individuals with comorbidity awareness and additional adjustment for dietary sodium intake (n=4429). 9](#_Toc100334212)

[**Supplementary Figure 5.** Sensitivity analysis 4: Model was adjusted for age, sex, TEI, municipality of residence, BMI, physical activity level, educational level, smoking habit; excluding individuals with comorbidity awareness and additional adjustment for fasting status (n=4429). 10](#_Toc100334213)

[**Supplementary Figure 6.** Sensitivity analysis 5: Model was adjusted for age, sex, TEI, municipality of residence, BMI, physical activity level, educational level, smoking habit; excluding individuals with comorbidity awareness and additional adjustment for each other source of protein (n=4429). 11](#_Toc100334214)

# **Supplementary Note 1**. Definition of additional exposure variables

Body mass index (BMI) was estimated as (weight, kg)/(height, m)^2^. Educational level, defined as the highest level of education completed, was classified as: up to lower-secondary level; professional qualification; upper-secondary education level; and university degree or higher. Physical activity during the last seven days was assessed by self-administration of the International Physical Activity Questionnaire (IPAQ) short version [1], and classified as low, moderate or high. Missing physical activity values (8%) were classified into a separate category to limit sample size loss. Reasons behind missingness were: items not completed by the participant; data entry errors; and values removed during data cleaning and processing according to IPAQ guidelines [2]. Smoking habits were assessed using the European Community Respiratory Health Survey II smoking questionnaire [3], with participants classified as never (never smoked or smoked for <1 year in the lifetime), past (smoked for ≥1 year but stop ≥1 month before the interview) and current smokers (currently smoking or stopped <1 month before the interview).

# **Supplementary Note 2**. The GA^2^LEN food frequency questionnaire (FFQ).

*Validation of the GA^2^LEN FFQ*

The GA^2^LEN FFQ was validated in a multinational sample of European adults and shown to be reliable to ascertain usual dietary intake of several foods [4, 5]. Assessment of protein intake showed an intraclass correlation coefficient (ICC) for repeatibility of 0.78 across countries, with a maximum of 0.86 in Germany [4], which shares the same written and spoken language as the vast majority of CHRIS study participants. Total energy intake (TEI) assessment had ICC=0.76 overall with a maximum of 0.89 in Germany [4]. Recently, the ability of the FFQ to identify flavonoid and polyphenolic intake was also assessed [5].

*Administration of the GA^2^LEN FFQ in the CHRIS study*

Participants were mailed home a paper-based version of the FFQ, asked to fill it in, and to bring it to the study centre on the day of the appointment. To limit data missingness, upon visual inspection, study assistants asked participants to complete any inadvertently skipped item. Questionnaires were scanned and responses captured by optical mark recognition (SDAPS program, <https://sdaps.org>).

# **Supplementary Note 3**. Additional details on statistical models and software

Linear mixed models were fitted the *lmekin* function in the R package ‘coxme’ v.2.2-16 (<https://cran.r-project.org/web/packages/coxme>) as previously described [6, 7]]. Relatedness was accounted for by estimating kinship coefficients from the genetic data using the package ‘kinship2’ v.1.8.5. To control for confounding effect of the time of enrollment, we included the week of examination as random effect in all models [6]. All statistical analyses were conducted in Stata v.15.1 (STATA StataCorp, College Station, TX, USA) and R v.3.5.2 ([www.R-project.org](http://www.R-project.org)).

**References**

1. Craig CL, Marshall AL, Sjöström M, et al (2003) International physical activity questionnaire: 12-country reliability and validity. Med Sci Sports Exerc 35:1381–95. https://doi.org/10.1249/01.MSS.0000078924.61453.FB

2. Guidelines for the data processing and analysis of the International Physical Activity Questionnaire. www.ipaq.ki.se. Accessed 23 Apr 2022

3. Chinn S, Jarvis D, Melotti R, et al (2005) Smoking cessation, lung function, and weight gain: a follow-up study. Lancet 365:1629–1635. https://doi.org/10.1016/S0140-6736(05)66511-7

4. Garcia-Larsen V, Luczynska M, Kowalski ML, et al (2011) Use of a common food frequency questionnaire (FFQ) to assess dietary patterns and their relation to allergy and asthma in Europe: pilot study of the GA2LEN FFQ. Eur J Clin Nutr 65:750–756. https://doi.org/10.1038/ejcn.2011.15

5. Charles D, Gethings LA, Potts JF, et al (2021) Mass spectrometry-based metabolomics for the discovery of candidate markers of flavonoid and polyphenolic intake in adults. Sci Rep 11:5801. https://doi.org/10.1038/s41598-021-85190-w

6. Noce D, Gögele M, Schwienbacher C, et al (2017) Sequential recruitment of study participants may inflate genetic heritability estimates. Hum Genet 136:743–757. https://doi.org/10.1007/s00439-017-1785-8

7. Murgia F, Melotti R, Foco L, et al (2019) Effects of smoking status, history and intensity on heart rate variability in the general population: The CHRIS study. PLoS One 14:e0215053. https://doi.org/10.1371/journal.pone.0215053

# **Supplementary Table 1**. Food items and the amounts of containing proteins.

Provided as Supplementary xlsx file

# **Supplementary Table 2.** Results of the linear mixed model fitting to assess the association between eGFR and ln-TDPI% and source-specific ln-DPI% (Model 3, n=4429).

| **Protein source, (ln-%energy)** | **b (95% CI), ml/min/1.73m^2^** |
| --- | --- |
| Total protein | -4.3 (-6.5, -2.1) |
| Animal protein | -2.4 (-3.5, -1.3) |
| Plant protein | 1.2 (-0.4, 2.7) |
| Combined sources | 0.1 (-1.3, 1.4) |
| Poultry | -1.4 (-2.2, -0.6) |
| Red, processed meat and offal | -1.4 (-2.1, -0.7) |
| Processed meat | -1.3 (-2.1, -0.5) |
| Fish | -1.9 (-2.7, -1.2) |
| Eggs | 0.1 (-1.4, 1.7) |
| Dairy | 0.3 (-0.5, 1.1) |
| Grains | 1.1 (-0.1, 2.3) |
| Legumes | 0.7 (-0.7, 2.1) |
| Other plants | -0.2 (-1.4, 1.1) |
| Abbreviations: b, coefficient of association; CI, confidence interval  Model 3 was adjusted for: age, sex, TEI and municipality of residence, BMI, physical activity level, educational level, and smoking habit; with exclusion of individuals with comorbidity awareness. | |

# ***Supplementary* Table 3.** Sensitivity analyses and corresponding results of the linear mixed model fitting to assess the association between eGFR and ln-TDPI% and source-specific ln-DPI%.

|  | **Sensitivity 1** | **Sensitivity 2** | **Sensitivity 3** | **Sensitivity 4** | **Sensitivity 5** |
| --- | --- | --- | --- | --- | --- |
| **Protein source, (ln-%energy)** | b (95% CI) | b (95% CI) | b (95% CI) | b (95% CI) | b (95% CI) |
| **Total protein** | -4.3 (-6.6, -2.1) | -4.1 (-6.2, -2.0) | -4.6 (-7.0, -2.3) | -4.4 (-6.6, -2.2) | - |
| **Animal protein** | -2.5 (-3.6, -1.3) | -2.2 (-3.3, -1.2) | -2.6 (-3.8, -1.4) | -2.4 (-3.5, -1.3) | -2.6 (-3.9, -1.3) |
| **Plant protein** | 1.0 (-0.6, 2.6) | 1.0 (-0.5, 2.5) | 1.1 (-0.5, 2.7) | 1.1 (-0.4, 2.7) | -0.5 (-2.3, 1.3) |
| **Combined sources** | 0.2 (-1.2, 1.7) | 0.1 (-1.2, 1.5) | 0.1 (-1.3, 1.5) | 0.1 (-1.3, 1.4) | -0.7 (-2.3, 0.8) |
| **Poultry** | -1.4 (-2.2, -0.6) | -1.3 (-2.1, -0.5) | -1.4 (-2.2, -0.6) | -1.4 (-2.2, -0.6) | -0.9 (-1.7, -0.1) |
| **Red/processed meat, offal** | -1.4 (-2.1, -0.7) | -1.3 (-2.0, -0.6) | -1.5 (-2.2, -0.7) | -1.4 (-2.1, -0.7) | -0.8(-1.7, 0.2) |
| **Processed meat** | -1.3 (-2.2, -0.5) | -1.3 (-2.1, -0.5) | -2.0 (-3.1, -0.8) | -1.3 (-2.2, -0.5) | -0.7 (-1.8, 0.4) |
| **Fish** | -2.0 (-2.8, -1.1) | -1.8 (-2.6, -1.0) | -1.9 (-2.7, -1.1) | -1.9 (-2.7, -1.1) | -1.8 (-2.6, -1.0) |
| **Eggs** | 0.3 (-1.3, 1.9) | 0.4 (-1.1, 1.9) | 0.1 (-1.4, 1.7) | 0.1 (-1.5, 1.7) | 0.6 (-1.0, 2.2) |
| **Dairy** | 0.3 (-0.5, 1.1) | 0.1 (-0.6, 0.9) | 0.4 (-0.4, 1.2) | 0.3 (-0.5, 1.1) | 0.1 (-0.8, 1.0) |
| **Grains** | 1.3 (0.0, 2.6) | 1.2 (0.1, 2.4) | 1.1 (-0.1, 2.3) | 1.1 (-0.1, 2.3) | 0.1 (-1.4, 1.5) |
| **Legumes** | 0.9 (-0.6, 2.4) | 0.2 (-1.2, 1.5) | 0.6 (-0.8, 2.1) | 0.6 (-0.8, 2.0) | 0.4 (-1.1, 1.9) |
| **Other plants** | -0.6 (-1.9, 0.7) | -0.5 (-1.7, 0.7) | -0.3 (-1.6, 1.0) | -0.2 (-1.4, 1.1) | -1.1 (-2.6, 0.4) |
| Abbreviations: b, coefficient of association; CI, confidence interval  Models were adjusted for: age, sex, TEI, municipality of residence, BMI, physical activity level, educational level, smoking habit; excluding individuals with comorbidity awareness and:  *Sensitivity analysis 1:* additionally removing participants currently on a special diet* (n=4238).  *Sensitivity analysis 2:* additionally removing participants with missing physical activity information (n=4080).  *Sensitivity analysis 3:* additional adjustment for dietary sodium intake (n=4429).  *Sensitivity analysis 4:* additional adjustment for fasting status (n=4429).  *Sensitivity analysis 5:* additional adjustment for each other source of protein (n=4429). | | | | | |

Supplementary Figure 1. Association between daily total protein intake and protein from different sources and age-and sex-adjusted eGFR levels (ml/min/1.73 m^2^).

**
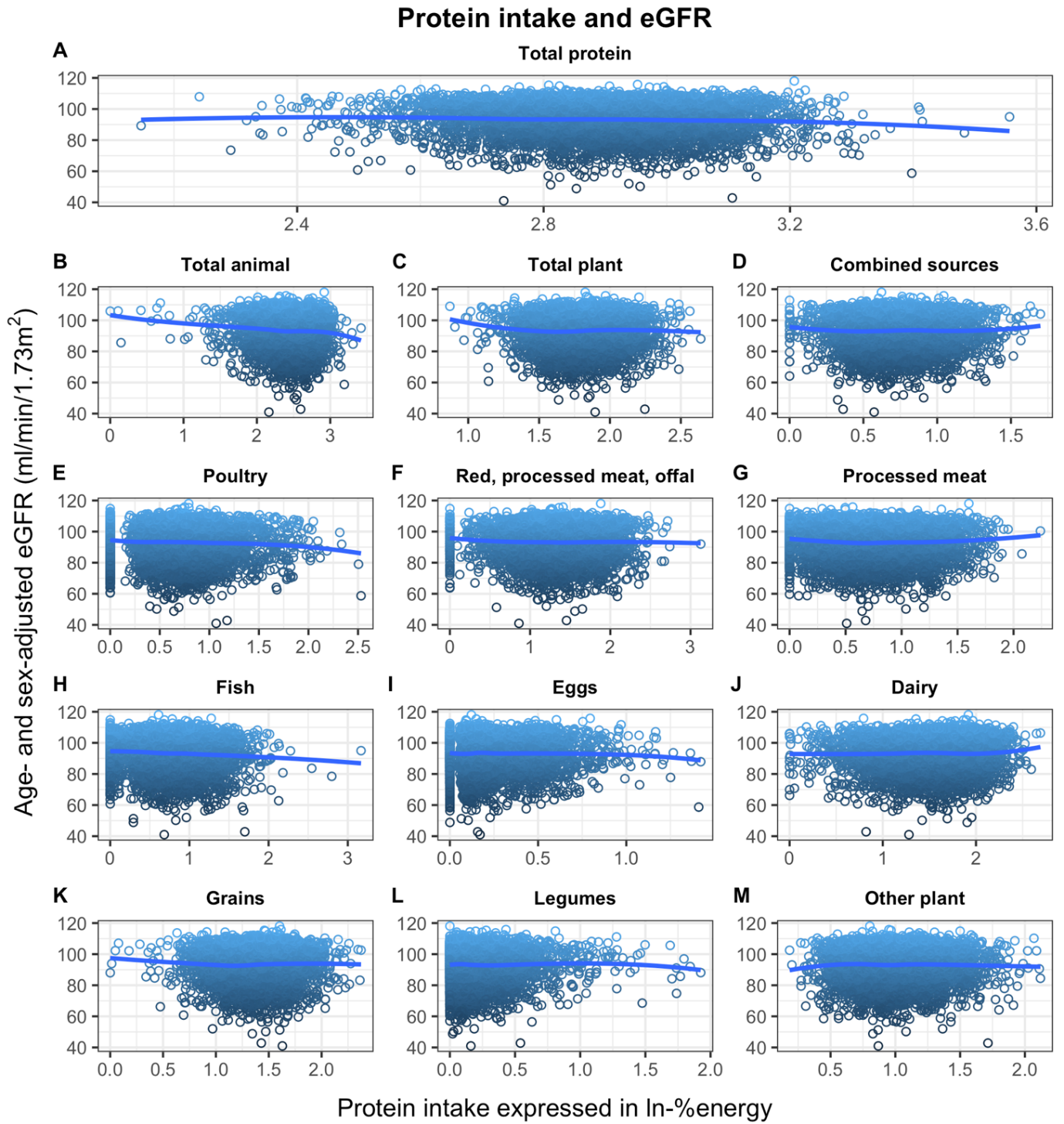
**

# **Supplementary Figure 2**. Sensitivity analysis 1: Model 3 (adjusted for age, sex, TEI, municipality of residence, BMI, physical activity level, educational level, smoking habit, and excluding individuals with comorbidity awareness), further excluding individuals on a special diet* (n=4238).


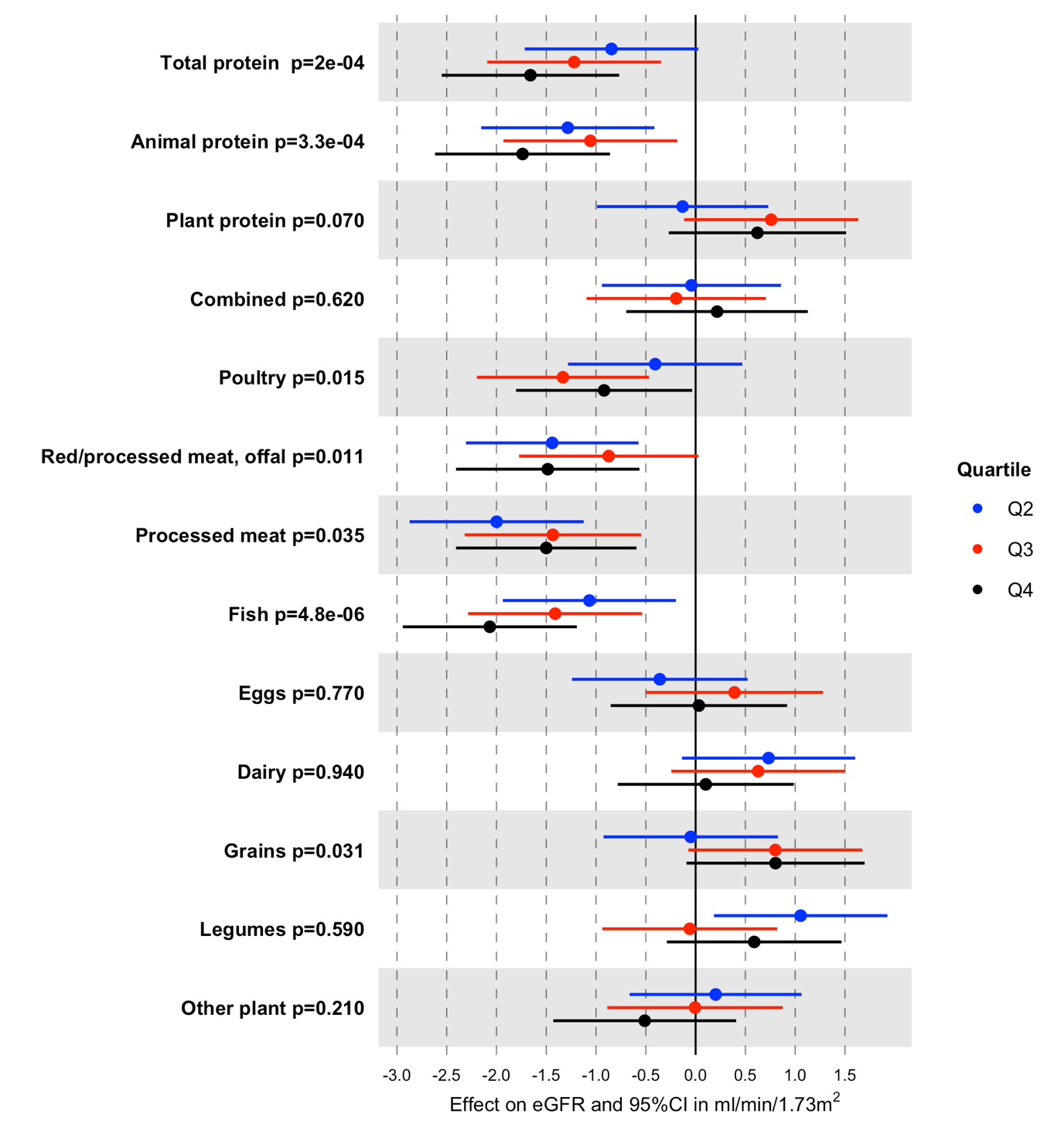


# **Supplementary Figure 3.** Sensitivity analysis 2: Model was adjusted for age, sex, TEI, municipality of residence, BMI, physical activity level, educational level, smoking habit; excluding individuals with comorbidity awareness and those with missing physical activity information (n=4080).


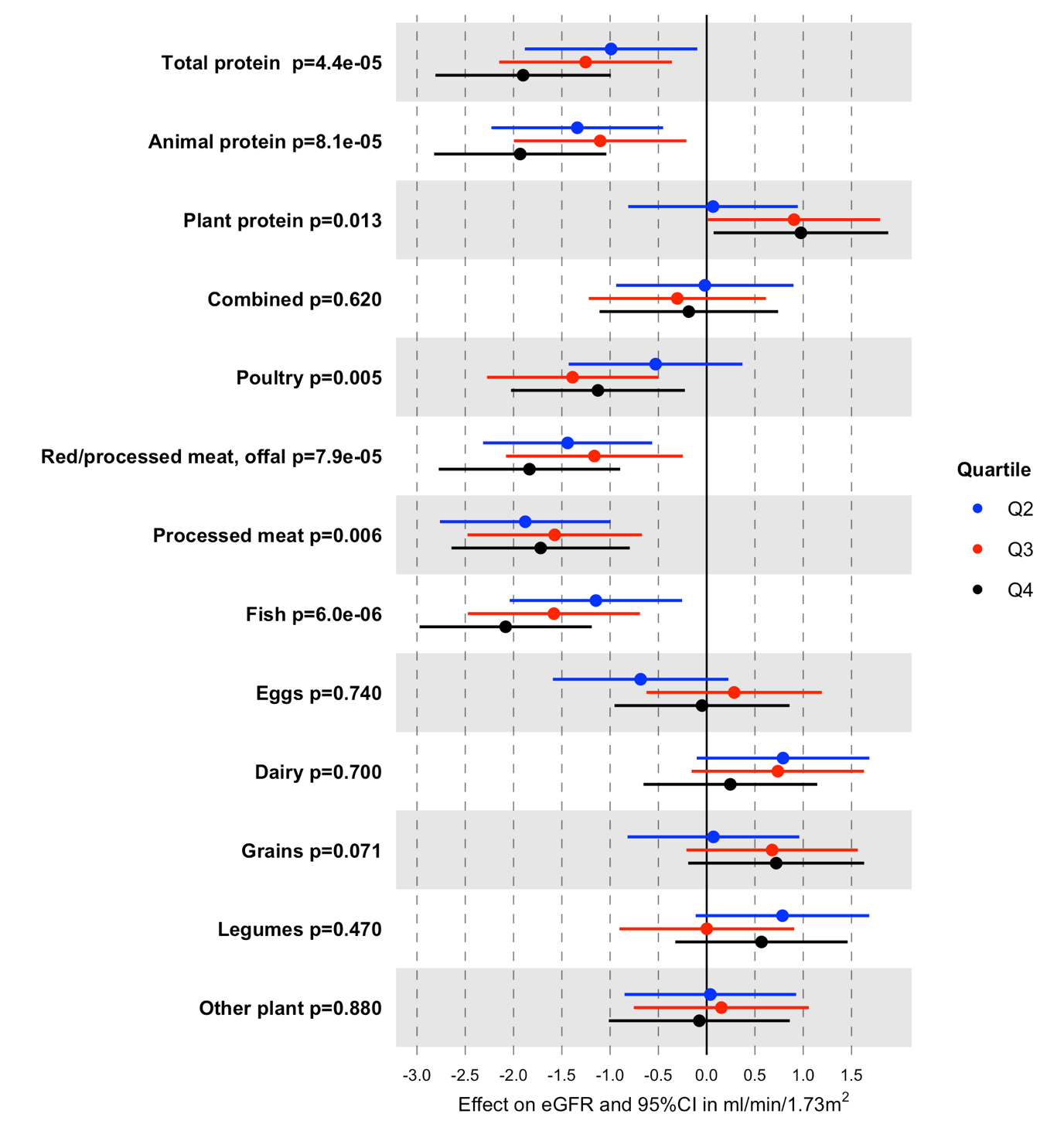


# **Supplementary Figure 4.** Sensitivity analysis 3: Model was adjusted for age, sex, TEI, municipality of residence, BMI, physical activity level, educational level, smoking habit; excluding individuals with comorbidity awareness and additional adjustment for dietary sodium intake (n=4429).


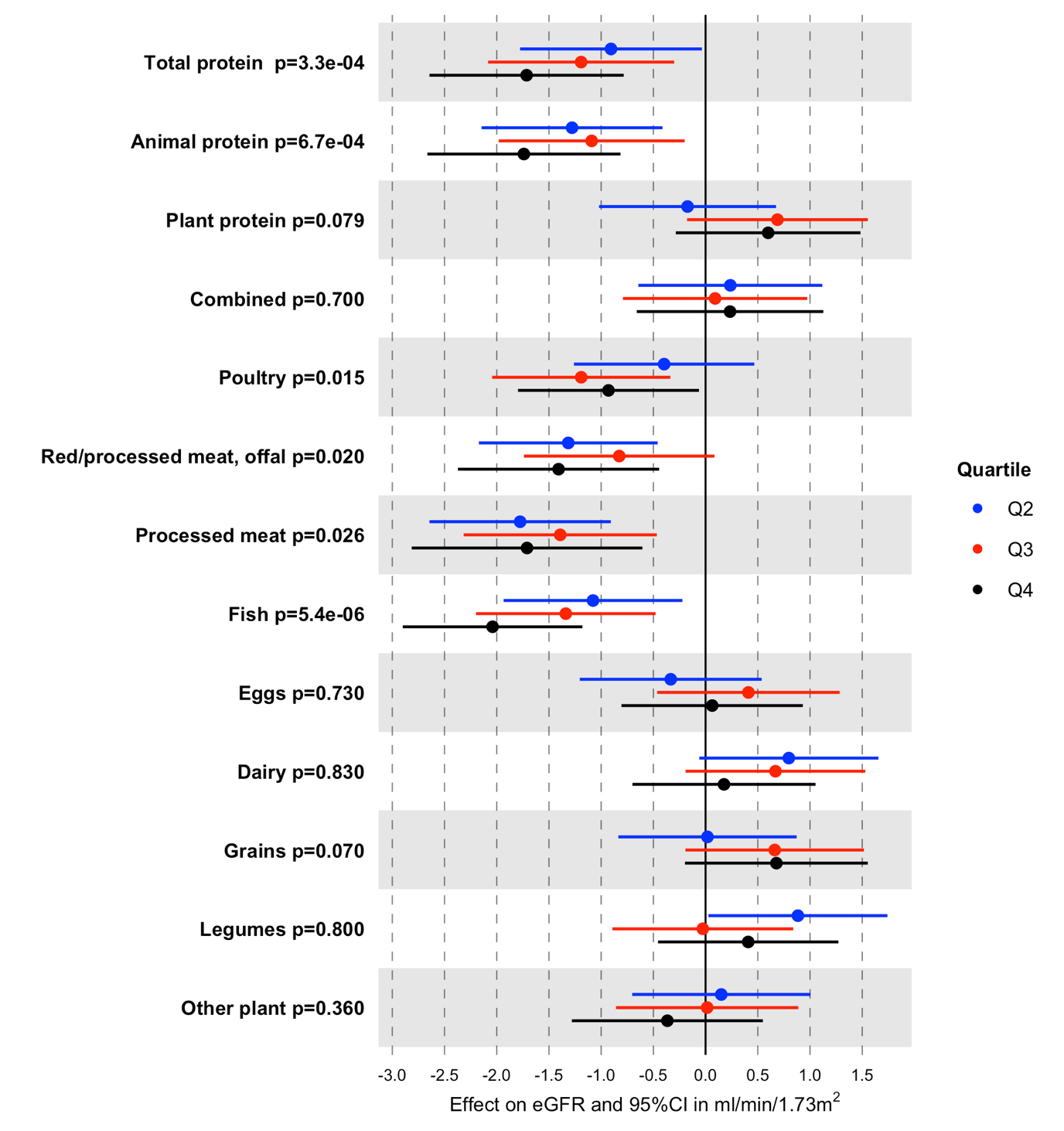


# **Supplementary Figure 5.** Sensitivity analysis 4: Model was adjusted for age, sex, TEI, municipality of residence, BMI, physical activity level, educational level, smoking habit; excluding individuals with comorbidity awareness and additional adjustment for fasting status (n=4429).


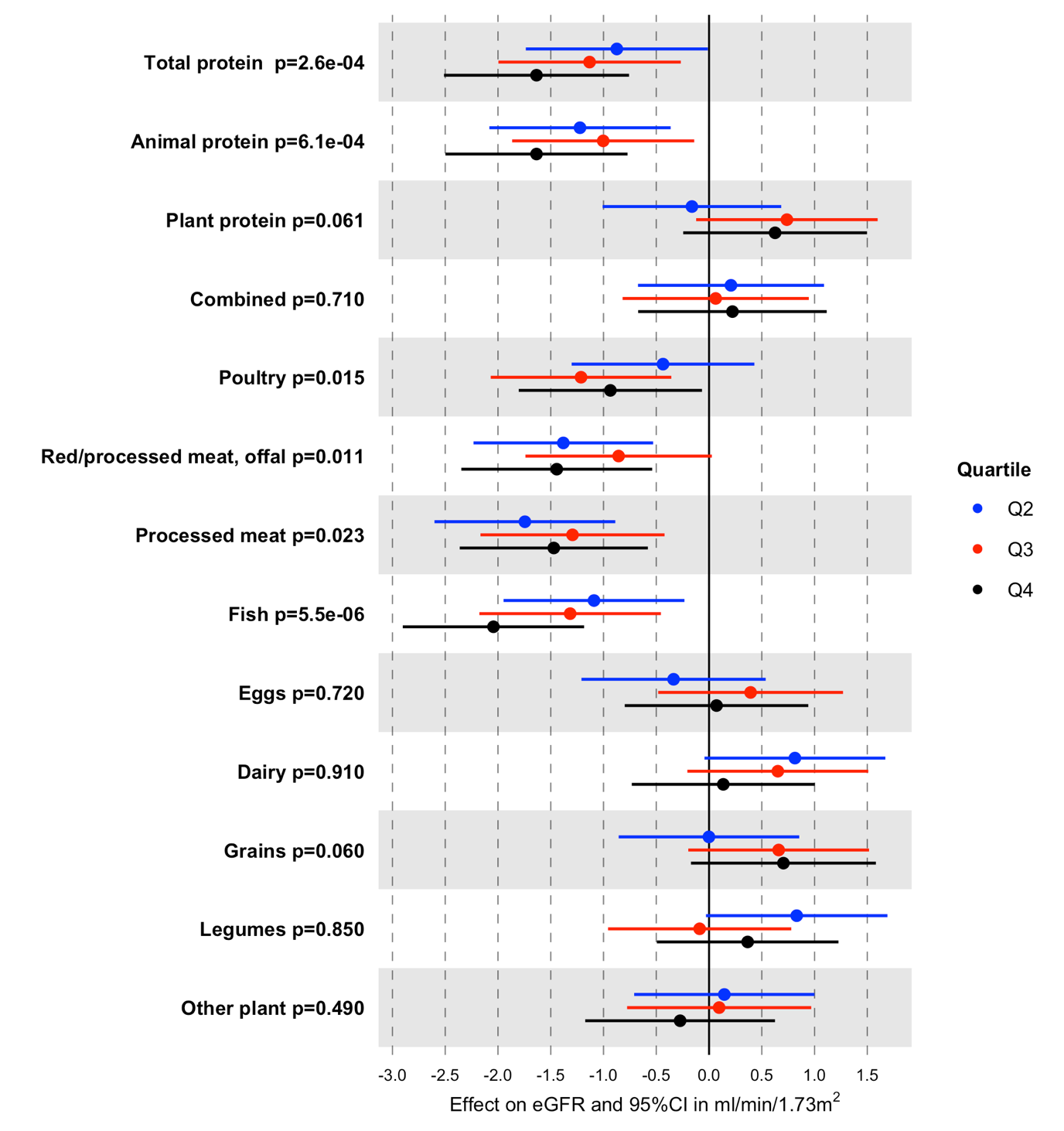


# **Supplementary Figure 6.** Sensitivity analysis 5: Model was adjusted for age, sex, TEI, municipality of residence, BMI, physical activity level, educational level, smoking habit; excluding individuals with comorbidity awareness and additional adjustment for each other source of protein (n=4429).


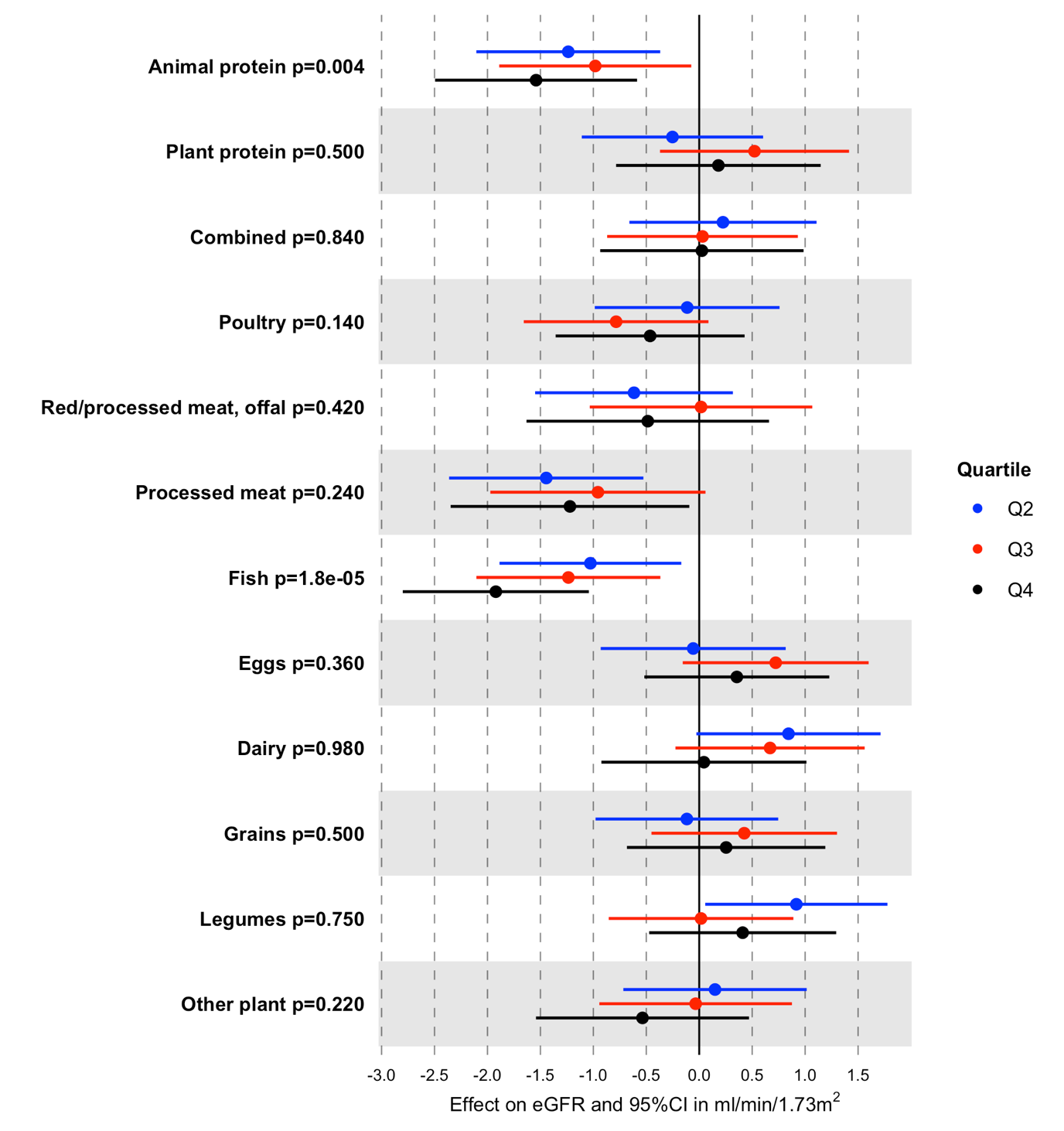

Supplement: Supplementary file 1 — Supplementary file1 (DOCX 2426 KB) [file 40620_2022_1409_MOESM1_ESM.docx]
